# Low CETP activity and unique composition of large VLDL and small HDL in women giving birth to small-for-gestational age infants

**DOI:** 10.1038/s41598-021-85777-3

**Published:** 2021-03-18

**Authors:** Marie Cecilie Paasche Roland, Kristin Godang, Pål Aukrust, Tore Henriksen, Tove Lekva

**Affiliations:** 1 National Advisory Unit for Women’s Health, Oslo University Hospital, Rikshospitalet, Oslo, Norway; 2 Department of Obstetrics, Oslo University Hospital, Rikshospitalet, Oslo, Norway; 3 Section of Specialized Endocrinology, Department of Endocrinology, Oslo University Hospital, Rikshospitalet, Oslo, Norway; 4 Research Institute of Internal Medicine, Oslo University Hospital, Rikshospitalet, Oslo, Norway; 5 Faculty of Medicine, University of Oslo, Oslo, Norway; 6 Section of Clinical Immunology and Infectious Diseases, Oslo University Hospital, Rikshospitalet, Oslo, Norway

**Keywords:** Endocrine system and metabolic diseases, Translational research

## Abstract

Cholesteryl ester transfer protein (CETP) regulates high density lipoproteins (HDL)-cholesterol (C) and HDL-C is essential for fetal development. We hypothesized that women giving birth to large-for-gestational-age (LGA) and small-for-gestational age (SGA) infants differed in longitudinal changes in lipoproteins, CETP activity and HDL-C and that placentas from women with higher or lower circulating HDL-C displayed differential expression of mRNAs involved in cholesterol/nutrient transport, insulin signaling, inflammation/ extracellular matrix (ECM) remodeling. Circulating lipids and CETP activity was measured during pregnancy, NMR lipidomics in late pregnancy, and associations with LGA and SGA infants investigated. RNA sequencing was performed in 28 placentas according to higher and lower maternal HDL-C levels. Lipidomics revealed high triglycerides in large VLDL and lipids/cholesterol/cholesteryl esters in small HDL in women giving birth to SGA infants. Placentas from women with higher HDL-C had decreased levels of *CETP* expression which was associated with mRNAs involved in cholesterol/nutrient transport, insulin signaling and inflammation/ECM remodeling. Both placental and circulating CETP levels were associated with growth of the fetus. Low circulating CETP activity at 36–38 weeks was associated with giving birth to SGA infants. Our findings suggest a link between increased maternal HDL-C levels, low CETP levels both in circulation and placenta, and SGA infants.

## Introduction

Being born too large (large-for-gestational age, LGA) or too small (small-for gestational-age, SGA) is associated with perinatal mortality and also with subsequent adult obesity, diabetes and cardiovascular disease for the child^1,2^. Fetal growth is a result of multiple factors including maternal nutrition and metabolism, the fetal genetic potential for growth and placental properties including its metabolism and endocrine function, blood perfusion and structural features. Previous clinical studies have shown a direct association between maternal plasma cholesterol levels and birthweight^3–5^.

Cholesterol and in particular high-density lipoprotein cholesterol (HDL-C) has essential roles in fetal development and in early gestation maternal cholesterol is transferred to the fetus across the placenta whereas in late pregnancy the fetus is able to synthesize its own cholesterol^6–8^. Importantly, more than 50% of fetal total cholesterol is carried by HDL^9^, which is different from the maternal circulation where low-density lipoprotein (LDL) represents the major class of lipoproteins. The maternal plasma cholesterol is taken up by the syncytiotrophoblast facing the maternal blood, followed by entrance into the subsyncytial endothelial cells of the fetoplacental vasculature wherefrom cholesterol is effluxed or secreted by specific transport proteins into the fetal circulation. The cholesterol transport may be mediated both by receptor-dependent and independent mechanisms besides that placenta may make its own cholesterol^10^.

HDL has diverse functions, including reverse cholesterol and lipid transport, role in inflammation through its anti-inflammatory properties, in hemostasis through its ability to modulate platelet function and seems also to be linked to insulin resistance. All these properties may indeed influence pregnancy outcomes. HDL function is related to the protein cargo carried by HDL (ApoA1, ApoA2, apoC, apoE, apoM, apoA4, lecithin–cholesterol acyltransferase (LCAT), cholesteryl ester transfer protein (CETP) and paraoxonase 1 (PON1)^11^. However, there is a difference in the protein cargo between fetal and maternal HDL, and proteins involved in lipid metabolism, inflammatory pathways and innate immunity were differentially expressed between the mother and the fetus^9^. Fetal and maternal HDL undergoes constant remodeling, that is dependent on the metabolic status of the mother and/or the fetus^12,13^. Inflammation is accompanied by changes in HDL lipid composition resulting in reduction of phospholipid and cholesteryl esters and an increase in triglycerides (TG)^14^.

Mice with apolipoprotein (apo) A1 deficiency, being the major protein in the HDL particles, displayed reduced fetal growth in dams with lower maternal cholesterol levels^15,16^. It has been hypothesized that the ability of the placenta to take up HDL-cholesterol and transport it to the fetus is compromised in women giving birth to SGA, and a reason for the high maternal HDL-cholesterol levels in these women^17^. Notably, even if the infant is synthesizing cholesterol, a lack of transfer of maternal cholesterol may impact growth^10^.

Cholesteryl ester transfer protein (CETP) is a glycoprotein that catalyzes the exchange of cholesteryl esters for TG between HDL and apolipoprotein B (apoB) containing lipoproteins and decreases HDL-C^18^. Increased TG content in the HDL particle may therefore be explained by increased CETP activity and CETP mediated replacement of cholesteryl esters by TGs in the HDL core results in lower plasma HDL-C levels. The composition of HDL and thereby partly its function during fetal growth is highly associated with CETP activity. To our knowledge there is no data in the literature of the function of CETP in the placenta. Interestingly data obtained from public repositories shows that CETP expression in the placenta is among the highest of all tissues, increases markedly during gestation and is increased in endothelial progenitor cells isolated from cord blood.

We hypothesized that placentas from women with higher and lower circulating maternal HDL-C displayed differential expression of mRNAs involved in cholesterol/nutrient transport, insulin signaling and inflammation/extracellular matrix (ECM) remodeling. We also hypothesized that women giving birth to LGA and SGA infants displayed differences in longitudinal changes in maternal lipids and CETP activity. To elucidate the role of HDL-C and CETP and their potential interaction in fetal growth we investigated in a prospective cohort study (STORK) (i) differences in longitudinal change in lipids (cholesterol including HDL-C and TG) in women giving birth to LGA and SGA infants, (ii) the RNA expression by RNA sequencing in 28 placentas from women divided into higher and lower maternal HDL-C circulating levels at week 36–38, (iii) *CETP* mRNA expression and its association with cholesterol/nutrient transport, insulin signaling and inflammation/ECM remodeling in the placenta and (iv) CETP activity in plasma measured multiple times during pregnancy of 300 women and its association with LGA and SGA babies, (v) lipoprotein composition in plasma of 160 women at week 36–38 and its association with LGA and SGA babies with particular focus of HDL particles.

## Methods

The STORK study is a prospective longitudinal cohort study in which 1031 low-risk women of Scandinavian heritage were followed throughout their pregnancy and gave birth at Oslo University Hospital Rikshospitalet between 2002 and 2008^19^. The exclusion criteria were multiple pregnancies, known pre-gestational diabetes and any severe chronic diseases (lung, cardiac, gastrointestinal or renal). Each pregnant woman had four study-related antenatal visits at weeks 14–16, 22–24, 30–32, and 36–38. A 75 g OGTT was performed on all women at 14–16 and 30–32 weeks of gestation. The follow-up study was performed 5-year after the index pregnancy^20^. Three hundred women agreed to participate and subjects included in this study are participants included in the follow-up study using samples from pregnancy (i.e. a sub-study of the STORK study). Blood samples were drawn in the morning between 07:30 and 08:30 after an overnight fast, centrifuged and stored at − 80 °C. EDTA plasma were stored on ice before centrifuged. Written informed consent was obtained from all study participants. All clinical investigations were conducted in accordance with the principles enshrined in the Declaration of Helsinki. The study was approved by the Regional Committee for Medical Research Ethics of Southern Norway in Oslo, Norway.

### AGA, LGA and SGA infants

Infants were divided according to birth weight: appropriate-for-gestational age (AGA), between 10 and 90th percentile, LGA, > 90th percentile and SGA < 10th percentile, adjusted for gestational age and fetal sex, according to Norwegian reference curves^21^.

### Intrauterine measurements of fetal growth

Ultrasound measurement was performed and head circumference, femur length and abdominal circumference (AC) at 22–24, 30–32, and 36–38 weeks was recorded as previously described^19^. The measurements were done by three people. The measurements of each participant were done by the same operator each time to minimize interobserver differences. All measurements were done three times and the average value was used.

### Lipoproteins and lipids

Lipoproteins and lipids were measured in the 1031 women at an accredited clinical chemistry laboratory by immunoassay (IMMULITE 2000; Siemens Healthcare GmbH, Erlangen, Germany) at Oslo University Hospital, Rikshospitalet^22^. Total cholesterol, HDL-C and triglycerides were measured at weeks 14–16 and 36–38 during pregnancy. LDL-C was determined by Friedewald's formula^23^. A commercially high–throughput proton NMR metabolomics platform (Nightingale Health Ltd., Helsinki, Fin) was used to quantify lipoprotein subclass profile with lipid concentrations, abundant proteins and various low-molecular-weight metabolites in fasting EDTA plasma at gestational weeks 36–38 in a subset of the women giving birth to 90 AGA, 48 LGA and 22 SGA infants. The subclass sizes were defined by their average diameter: extremely large (XXL) very low density lipoprotein (VLDL) chylomicrons (> 75 nm), extra-large (XL), large (L), medium (M), small and extra-small (XS) VLDL (64.0, 53.6, 44.5, 36.8, and 31.3 nm), intermediate lipoprotein IDL (28.6 nm), L, M, and S LDL subclasses (25.5, 23.0 and 18.7 nm), and XL, L, M and S HDL subclasses (14.3, 12.1, 10.9 and 8.7 nm). The components phospholipids (PL), cholesterol, cholesteryl esters (CE), free cholesterol (FC) and TG of the lipoprotein subclasses were quantified. The mean size for VLDL, LDL and HDL particles was calculated by weighting the corresponding subclass diameters with their particle concentrations.

### Measurement of CETP activity

Plasma CETP activity was measured in duplicate using commercially available kit (MAK106) from Sigma-Aldrich (St. Louis, MO), as previous published^24^. The reaction mixture contained a donor molecule that was a fluorescent self-quenching neutral lipid as well as an acceptor molecule. Five µL of diluted plasma sample was added to the reaction mixture and incubated for 3 h at 37 °C in a black 384 well plate. CETP-mediated transfer from donor to acceptor resulted in an increase in fluorescence intensity with an excitation wavelength of 465 nm and emission of 535 nm as read by the fluorescent plate reader. The CV for the analysis was < 13%. All 4 samples from one person were analyzed on the same plate.

### Collection, storage and RNA extraction of placental biopsies

Blocks of 2–4 cm were taken from the placental parenchyma, briefly washed in phosphate buffer saline, snap frozen in liquid nitrogen, and stored in − 80 °C until homogenization. Half of the biopsy was homogenized in TRIzol reagent (Invitrogen, Life Technologies) on ice with a tissue grinder (Sigma Aldrich). Total RNA was extracted using TRIzol reagent (Invitrogen, Life Technologies) and purified with RNeasy microkit columns (Qiagen, Netherlands) as previously published^25^. The electropherograms from the bioanalyzer and RIN values showed satisfying RNA quality, as previously published^25^. Samples chosen for RNAseq were of satisfying quality with a small percentage of contamination by maternal decidual and nucleated blood cells (~ 2%). Furthermore, the most abundantly expressed genes identified in the term placenta were ones known to be involved in placental function, with good overlap with transcripts identified in previous studies.

### RNAseq

Samples were selected as previously described^25^. Sequencing libraries were prepared from 500 ng of total RNA using the TruSeq RNA sample preparation reagents (Illumina, San Diego, CA) according to the manufacturer’s instructions, with fragmentation for 4 min at 94 °C. The libraries were sequenced using 125 bp paired-end sequencing on an Ilumina HiSeq 2000. We recorded an average 22.3 million (range, 20.2–24.6 million) paired reads per sample. Fastq files were generated using bcl2fastq (v1.8.4). Sequence reads were mapped to the reference genome (hg19/GRCh38) using TopHat2 (v2.0.13) and Bowtie2 (v.2.2.3.0). Library sizes and standard deviations for input into TopHat were calculated empirically by aligning 1,000,000 reads to an index built from human cDNA sequence. Sequence alignment was guided using only previously annotated gene models downloaded from Ensembl (http://www.ensembl.org; Homo_sapiens.GRCh38.79.gtf). On average, there was 73.6% concordant read pair mapping (range 69.6–76.6), with a mean unique mapping of 94.1%. Raw expression counts were calculated per gene using featureCounts (http://bioinf.wehi.edu.au/featureCounts/) and the same gtf file which was used for the read alignment. Differential expressed genes in RNAseq data was tested using DESeq2^26^ package for R dividing the samples between median HDL-C levels from the 1031 samples at 36–38 weeks of gestation. Outlier detection (Cook distance cutoff) and filtering out low expressed genes was performed using the default method in DESeq2, as previously published^25^.

### Statistical analysis

Statistical analyses were conducted using SPSS for Windows, version 21.0 (Chicago, IL, USA). In general, data are expressed as mean ± SD when normally distributed and median (25th, 75th percentile) when skewed. Differences in the temporal course of CETP, HDL-C, LDL-C and TG during pregnancy between birth weight groups was evaluated with repeated measures ANOVA a priori, and with Bonferroni adjusted *t*-tests between different groups a posteriori. Data from this analysis are expressed as estimated marginal means and 95% confidence intervals. The birth weight groups (SGA, LGA and AGA infants), birth weight and abdominal circumference (AC) were adjusted for gestational age and fetal sex. Associations between CETP expression/CETP activity and AC/delta AC and mRNA transcripts were evaluated by Spearman correlation. The association between CETP activity and AC/delta AC were also adjusted in linear regression models by age, BMI, fetal sex, gestational diabetes (GDM), and preeclampsia. Unadjusted and adjusted (age, BMI, fetal sex, GDM and preeclampsia) logistic regression model were performed on the CETP activity data comparing SGA vs AGA. Linear regression models were used on the lipidomics data comparing AGA with SGA/ LGA infants adjusting for age, fetal sex, GDM, preeclampsia and BMI at weeks 14–16 with adjustment for multiple comparisons, and data presented as standardized estimated marginal means and 95% confidence interval. Two-tailed p-values < 0.05 were considered significant, except for interactions analysis where p < 0.01 was considered significant. The RNAseq results were presented as log2fold and unadjusted p-values are presented.

## Results

Table 1 shows the characteristic of the study population including a total of 1031 women with 810 AGA, 142 LGA and 76 SGA newborns. In the total cohort the women giving birth to LGA infants were taller, had a longer duration of pregnancy, were less frequently primipara, gave birth to boys more frequent, had a higher BMI at 14–16 weeks and had a lower HDL-C at 36–38 weeks, compared to AGA. The women giving birth to SGA infants were shorter, had a shorter pregnancy duration, were more frequently primipara, gave birth to girls more frequent, had a lower BMI at 14–16 weeks and had a lower LDL-C at 36–38 weeks, compared to AGA. In the sub-study cohort of 300 women we found the same pattern except for the lack of differences in height, gestational age and LDL-C for SGA compared to AGA infants.

**Table 1:** Characteristics of the study population.

	Total	AGA infants	LGA infants	SGA infants
N	1031	810	142	76
Age (years)	31.2 ± 3.9	31.2 ± 3.9	31.8 ± 3.6	31.4 ± 3.6
Height (cm)	169 ± 6	169 ± 6	170 ± 6**	167 ± 6**
Gestational age (week)	39.9 ± 1.8	39.9 ± 1.7	40.5 ± 1.2***	39.1 ± 2.7**
Primipara, n (%)	545 (53)	441 (55.1)	45 (31.7)***	59 (77.6)***
BMI 14–16 week (kg/m^2^)	23.8 (21.8, 26.3)	23.6 (21.6, 26.1)	25.4 (23.8, 27.4)***	22.5 (20.7, 24.6)**
Birth weight (g)	3587 ± 575	3525 ± 422	4435 ± 267***	2674 ± 469***
Preterm, n (%) < 34/34–37 weeks	18 (1.7)/38 (3.7)	13 (1.6)/30 (3.7)	0/0*	5 (6.6)/7 (9.2)**
Preeclampsia, n (%)	38 (3.7)	26 (3.2)	8 (5.6)	4 (5.3)
Gestational diabetes^a^, n (%)	244 (24.8)	181 (23.3)	54 (40.0)***	9 (12.7)*
Male newborns, n (%)	548 (53.3)	417 (51.5)	107 (75.4)***	24 (31.6)**
Total C 14–16 weeks (mmol/l)	4.9 (4.3–5.4)	4.9 (4.4–5.5)	4.8 (4.3–5.4)	4.9 (4.2–5.3)
LDL-C 14–16 weeks (mmol/l)	2.52 (2.07–3.02)	2.53 (2.08–3.04)	2.51 (2.14–3.02)	2.45 (1.81–2.83)
HDL-C 14–16 weeks (mmol/l)	1.8 (1.5–2.1)	1.8 (1.5–2.1)	1.7 (1.5–2.0)	1.8 (1.5–2.2)
TG 14–16 weeks (mmol/l)	1.09 (0.89–1.39)	1.09 (0.89–1.38)	1.10 (0.88–1.46)	1.06 (0.88–1.41)
Total Chol 36–38 weeks (mmol/l)	6.7 (5.9–7.5)	6.7 (5.9–7.5)	6.5 (5.5–7.6)	6.6 (5.5–7.4)
LDL-C 36–38 weeks (mmol/l)	3.79 (3.15–4.52)	3.81 (3.20–4.52)	3.68 (2.87–4.77)	3.67 (2.75–4.13)*
HDL-C 36–38 weeks (mmol/l)	1.7 (1.4–2.0)	1.7 (1.4–2.0)	1.5 (1.3–1.8)***	1.8 (1–4-2.2)
TG 36–38 weeks (mmol/l)	2.37 (1.92–2.91)	2.35 (1.89–2.90)	2.45 (1.97–3.01)	2.29 (1.81–2.79)
CETP activity, N	300	227	49	23
Age (years)	32.2 ± 3.9	32.3 ± 4.0	32.4 ± 3.3	31.6 ± 3.0
Height (cm)	169 ± 6	169 ± 6	171 ± 6*	167 ± 6
Gestational age (wk)	40.0 ± 1.5	40.0 ± 1.5	40.5 ± 1.2*	40.0 ± 2.1
Primipara, n (%)	150 (50.0)	116 (51.8)	16 (32.7)*	18 (78.3)***
BMI 14-16w (kg/m^2^)	23.6 (21.6, 26.0)	23.6 (21.7, 25.8)	25.3 (22.6, 27.1)**	21.2 (20.3, 23.2)***
Birth weight (g)	3631 ± 539	3553 ± 375	4405 ± 258***	2740 ± 365***
Preterm, n (%) < 34/34–37 weeks	1 (0.3) / 12 (4.0)	0 (0) / 12 (5.3)	0 (0)/0 (0)	1 (4.3) / 0 (0)**
Preeclampsia, n (%)	10 (3.3)	6 (2.6)	1 (2.0)	3 (13)**
Gestational diabetes^a^, n (%)	72 (24.5)	53 (23.7)	18 (38.3)*	1 (4.5)*
Male newborns, n (%)	155 (51.8)	115 (50.7)	33 (67.3)*	7 (30.7)
Total C 14–16 weeks (mmol/l)	4.8 (4.3–5.4)	4.8 (4.2–5.4)	4.8 (4.3–5.3)	5.0 (4.6–5.3)
LDL-C 14–16 weeks (mmol/l)	2.42 (2.00–3.96)	2.41 (1.99–2.95)	2.48 (2.15–3.10)	2.50 (2.00–2.93)
HDL-C 14–16 weeks (mmol/l)	1.8 (1.6–2.1)	1.8 (1.6–2.1)	1.7 (1.5–2.0)	1.8 (1.7–2.2)
TG 14–16 weeks (mmol/l)	1.04 (0.85–1.32)	1.04 (0.84–1.30)	1.04 (0.87–1.39)	1.06 (0.82–1.33)
Total Chol 36–38 weeks (mmol/l)	6.6 (5.9–7.5)	6.6 (5.9–7.5)	6.4 (5.6–7.5)	6.8 (6.1–7.8)
LDL-C 36–38 weeks (mmol/l)	3.81 (3.03–4.66)	3.84 (3.05–4.66)	3.76 (3.10–4.75)	3.54 (2.89–4.63)
HDL-C 36–38 weeks (mmol/l)	1.7 (1.4–2.0)	1.7 (1.4–2.0)	1.5 (1.3–1.9)*	2.0 (1.5–2.3)
TG 36–38 weeks (mmol/l)	2.28 (1.77–2.76)	2.20 (1.76–2.71)	2.37 (1.88–2.88)	2.33 (1.91–2.79)
Placenta RNAseq, N	28	20	8	
Age (years)	31.7 ± 4.5	30.8 ± 4.5	33.9 ± 3.6	
Height (cm)	169 ± 6	169 ± 6	170 ± 6	
Gestational age (wk)	39.7 ± 0.8	39.8 ± 0.9	39.9 ± 0.7	
Primipara, n (%)	13 (46.4)	11 (55.0)	2 (25.0)	
BMI 14–16 weeks (kg/m^2^)	25.1 (22.0, 30.2)	25.1 (20.7, 30.2)	26.1 (22.6, 30.6)	
Birth weight (g)	3737 ± 504	3475 ± 295	4391 ± 243***	
Preterm, n (%) < 34/34–37 weeks	0 (0)	0 (0)	0 (0)	
Preeclampsia, n (%)	10 (35.7)	7 (35.0)	3 (37.5)	
Gestational diabetes^a^, n (%)	13 (46.4)	9 (45.0)	4 (50.0)	
C-sections, n (%)	8 (28.6)	5 (25.0)	3 (37.5)	
Male newborns, n (%)	15 (53.6)	9 (45.0)	6 (75.0)	
Total C 14–16 weeks (mmol/l)	5.0 (4.6–5.8)	4.9 (4.2–6.3)	5.1 (4.8–5.5)	
LDL-C 14–16 weeks (mmol/l)	2.53 (1.95–3.41)	2.55 (1.95–3.66)	2.46 (1.88–3.05)	
HDL-C 14–16 weeks (mmol/l)	1.8 (1.6–2.4)	1.6 (1.5–2.4)	2.3 (1.8–2.5)	
TG 14–16 weeks (mmol/l)	1.22 (0.79–1.62)	1.22 (0.78–1.69)	1.16 (0.90–1.38)	
Total Chol 36–38 weeks (mmol/l)	6.5 (5.9–8.6)	6.4 (5.9–9.0)	6.6 (5.8–7.9)	
LDL-C 36–38 weeks (mmol/l)	3.82 (2.75–5.02)	3.91 (2.81–5.83)	3.59 (2.48–4.79)	
HDL-C 36–38 weeks (mmol/l)	1.6 (1.4–2.1)	1.5 (1.4–2.3)	2.0 (1.7–2.1)	
TG 36–38 weeks (mmol/l)	2.52 (2.09–3.41)	2.54 (2.09–3.44)	2.41 (1.35–3.32)	

*AGA* appropriate-for-gestational age, *LGA* large-for-gestational age, *SGA* small-for-gestational age, *C* cholesterol, *TG* triglycerides. ^a^WHO2013, with representative OGTT. Data given as mean ± SD when normal distributed and median (25th, 75th) when skewed distributed. *p < 0.05, **p < 0.01, ***p < 0.001.

### HDL-C circulating levels are changing differently in pregnancy in mothers giving birth to LGA and SGA infants

**Figure 1:**
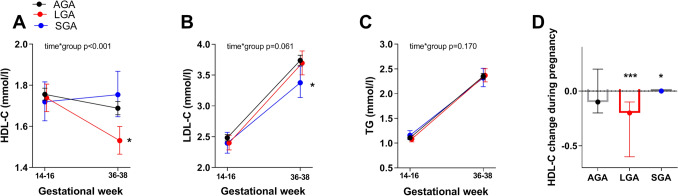
(**A–C**) Cholesterol and triglyceride levels at week 14–16 and week 36–38 during pregnancy between AGA (appropriate for gestational age) n = 731, LGA (large for gestational age) n = 135 and SGA (small for gestational age) n = 64 infants, adjusted for age and BMI at week 14–16. Data is given as estimated marginal means and 95% CI. (**D**) HDL change during pregnancy between AGA, LGA and SGA infants. Data is given as median (25th, 75th). *p < 0.05, ***p < 0.001 different from AGA infants.

Dividing our cohort according to birthweight into AGA, LGA and SGA infants, and adjusting for age and BMI, we found an interaction between time and group and a significant lower HDL-C at 36–38 weeks in women giving birth to LGA infants (Fig. 1A). Also, mothers giving birth to SGA infants had lower levels of LDL-C (Fig. 1B) at 36–38 weeks, while no difference in TG (Fig. 1C) was found between groups. As shown in Fig. 1D the change of HDL-C during pregnancy was significantly different for both LGA and SGA infants compared to AGA infants with a larger decrease in HDL-C in women giving birth to LGA infants, but not in women giving birth to SGA infants, compared to AGA infants.

### CETP mRNA levels are lower in placentas from women with the highest HDL-C levels at 36–38 weeks

**Figure 2:**
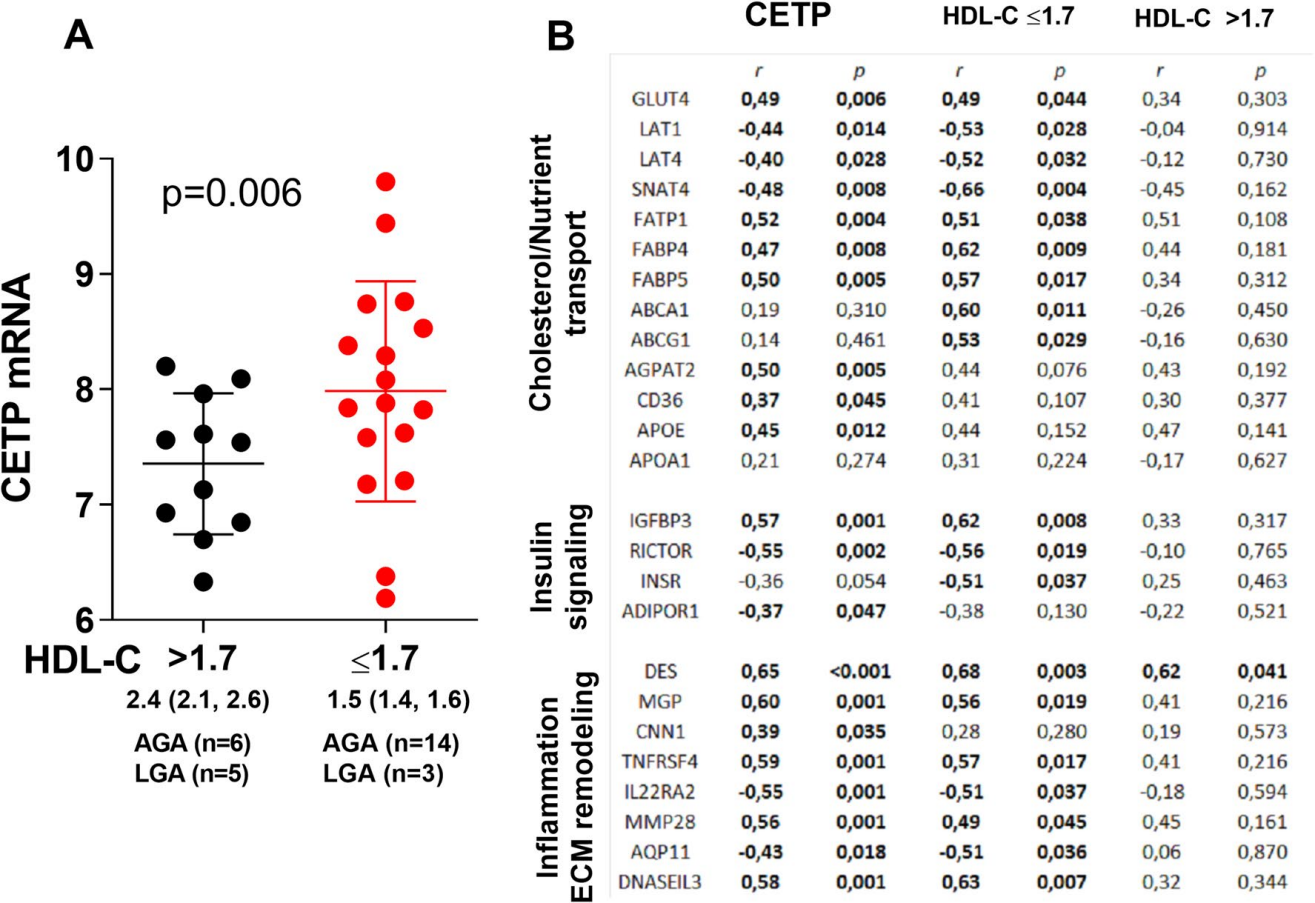
(**A**) CETP mRNA expression in the term placentas (n = 28) of mothers with circulating higher and lower HDL-C (median (25th, 75th) at week 36–38, p-value from RNAseq analysis. (**B**) CETP mRNA expression and associations with mRNAs involved in nutrient transport, insulin signaling and inflammation/ECM remodeling in the total 28 placentas, and divided in placentas from mothers with circulating higher and lower HDL-C at 36–38 weeks. Numbers represent correlation coefficient (r) and p-value (p).

As a model to investigate the possible effects or consequence of the maternal circulating HDL-C level on mRNAs in the placenta, we divided HDL-C levels from the 1031 women at week 36–38 weeks by the median, and used the cut-off to investigate the differentially expressed mRNAs from the RNAseq of 28 placenta biopsies (Table 1, Supplementary File). Many interesting mRNAs were regulated in relation to HDL-C levels including those involved in lipid metabolism and inflammation/ECM remodeling (Table 2). Importantly, *CETP* was one of the top-regulated mRNAs on the list and was decreased in the placentas from the group of mothers with the highest HDL-C levels (Fig. 2A). We found associations between *CETP* mRNA in the placenta and factors important for growth of the fetus; glucose transport [*glucose transport protein 4 *(*GLUT4*),* insulin receptor *(*INSR*)], amino acid transport [*L-amino acid transporter*(*LAT*)* 1 and 3, System A amino acid transporter 3 *(*SNAT4*)], fatty acid transport [*fatty acid binding protein *(*FABP*)* 4 and 5, fatty acid transport protein 1 *(*FATP1*), *CD36*, cholesterol transport (*ATP binding cassette subfamily A member 1 *(*ABCA1*)*, and G1 *(*ABCG1*)] and inflammation/ECM remodeling [*desmin* (*DES*),* matrix Gla protein *(*MGP*),* matrix metallopeptidase 28 *(*MMP28*),* TNF receptor superfamily, member 4 *(*TNFRSF4*),* interleukin 22 receptor subunit alpha 2 *(*IL22RA2*)]. We found strong associations with *CETP* in all these pathways (Fig. 2B). Comparing mRNAs from LGA vs AGA infants (Table 1, Supplementary File) we found no group differences in the mRNAs regulated in the higher and lower HDL-C groups. Nonetheless, the fact that down-regulation of CETP was strongly associated with high HDL-C levels could suggest a role for HDL-C in the regulation of all these genes.

**Table 2:** Some of the mRNAs involved in lipid metabolism and inflammation/ECM remodeling from the RNAseq analysis showing downregulated and upregulated mRNAs in placenta of women with the highest HDL-C levels.

mRNA	Name	p-value	Log2fold
**Lipid metabolism**			
AGPAT2	1-Acylglycerol-3-phosphate *O*-acyltransferase 2	0.002	− 0.61
PLA2G2A	Phospholipase A2 group IIA	0.025	− 0.62
RBP4	Retinol binding protein 4	0.003	0.97
APOL4	Apolipoprotein L4	0.001	0.68
CETP	Cholesteryl ester transfer protein	0.006	− 0.70
APOE	Apolipoprotein E	0.059	− 0.37
**Inflammation/ECM remodeling**			
DES	Desmin	< 0.001	− 0.72
MMP9	Matrix metallopeptidase 9	0.004	− 0.74
COMP	Cartilage oligomeric matrix protein	0.034	− 0.68
PTX3	Pentraxin 3	0.037	− 0.67
MGP	Matrix Gla protein	0.005	− 0.63
MMP28	Matrix metallopeptidase 28	0.013	− 0.61
CNN1	Calponin 1	0.010	− 0.70
IL22RA2	Interleukin 22 receptor subunit alpha 2	0.012	0.68
TNFRSF4	Tumor necrosis factor receptor superfamily, member 4	0.008	− 0.65
PRELP	Proline and arginine rich end leucine rich repeat protein	0.015	− 0.52
**Fetal HDL proteome**			
IGHG1	Immunoglobulin heavy constant gamma 1	< 0.001	1.07
IGLC2	Immunoglobulin lambda constant 2	< 0.001	1.26

### CETP mRNA and circulating levels are associated with abdominal circumference of the fetus

**Figure 3:**
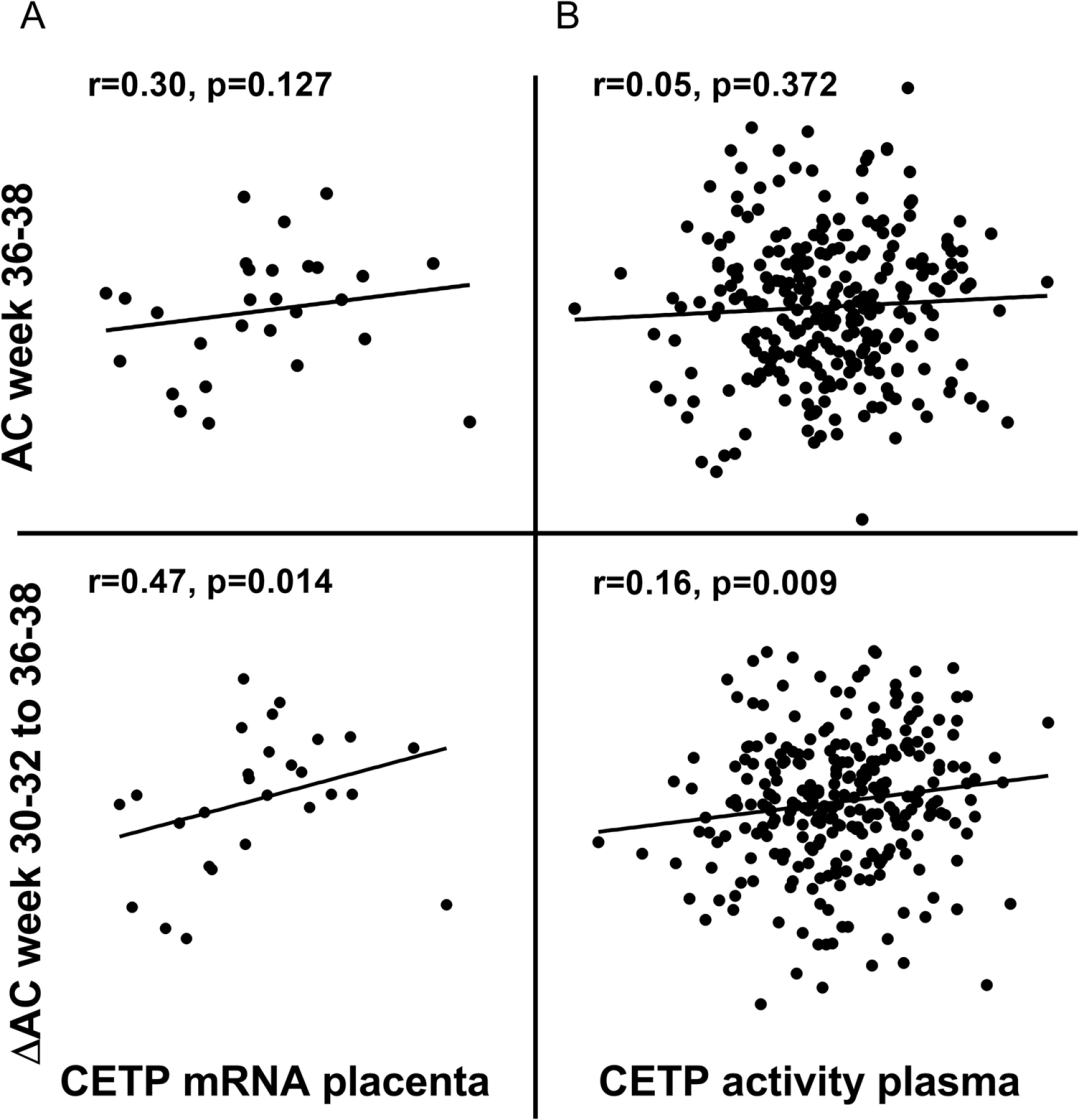
Correlation plot between (**A**) CETP mRNA in the placenta and abdominal circumference (AC) and the difference in AC between week 30–32 to 36–38, (**B**) CETP activity in plasma and AC and the difference between AC from week 30–32 to 36–38.

Our findings from the RNAseq analyses of placenta showed a down-regulation of *CETP* in the placenta of women with the highest HDL-C levels, suggesting an interaction between HDL-C and *CETP*, of which expression of the latter was associated with pathways involved in glucose and lipid metabolism as well as inflammation. To further elucidate these issues, we next investigated how the intrauterine fetal growth was associated with *CETP* expression in placenta and plasma. Abdominal circumference (AC) of the fetus at 36–38 week and the difference in AC from week 30–32 to 36–38, both adjusted for gestational age and sex, were associated with *CETP* mRNA expression in the placentas (r = 0.30, p = 0.127 and r = 0.47, p = 0.014), respectively; n = 28. Adjusting the associations in a linear regression model with age and BMI did not change the results, p = 0.084 and p = 0.036, respectively. Circulating CETP activity at 36–38 weeks in the women was not associated with AC at week 36–38, but associated with the difference in AC from week 30–32 to 36–38, (r = 0.16, p = 0.009, n = 278) (Fig. 3). Adjusting the associations in a linear regression model with age and BMI did not change the results, neither did adjusting with gestational diabetes, preeclampsia or fetal sex (data not shown). No associations were found between *CETP* and head circumference and fetal length.

### CETP circulating levels are lower in pregnant women giving birth to SGA infants

**Figure 4:**
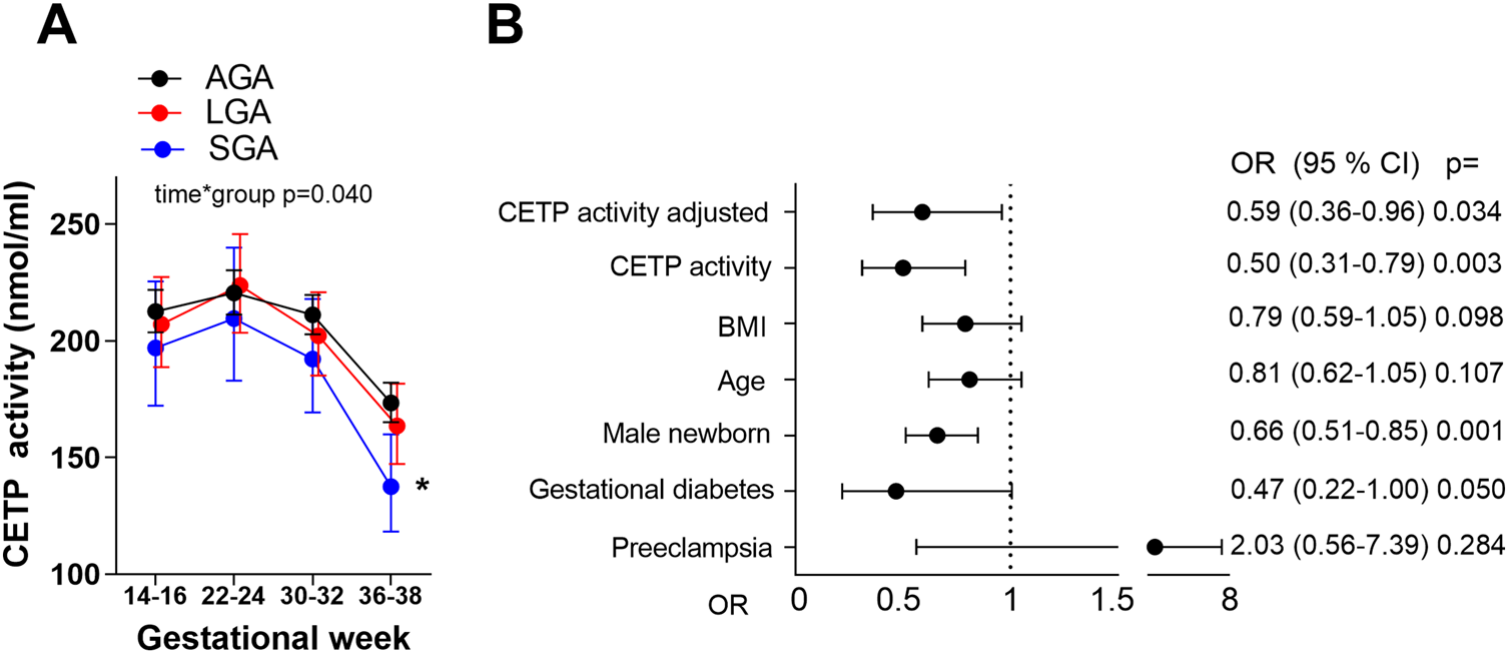
(**A**) CETP activity during pregnancy in women giving birth to AGA (appropriate for gestational age) n = 208, LGA (large for gestational age) n = 45 and SGA (small for gestational age) n = 22 infants, adjusted for age and BMI at week 14–16. *p < 0.05 between SGA and AGA infants. (**B**) Logistic regression showing univariate and adjusted (age, BMI, fetal sex, gestational diabetes and preeclampsia) models giving birth to SGA infants compared to AGA infants, odds ratio (OR), when the CETP activity is low at 36–38 weeks, using standardized values.

Analyzing the circulating CETP activity in women during pregnancy in 300 women divided by their birth of AGA, LGA and SGA infants, we found a lower CETP activity at 36–38 weeks in the women giving birth to SGA infants (Fig. 4A). Performing a logistic regression investigating CETP activity at 36–38 weeks comparing SGA vs. AGA infants, and adjusting for age, fetal sex, GDM, preeclampsia and BMI, we found almost a two times higher risk of giving birth to SGA infants when the CETP activity at 36–38 weeks decreased 1 log per standard deviation (Fig. 4B). However, CETP activity at week 36–38 did not predict SGA infants compared to LGA infants or predict LGA infants compared to AGA infants (data not shown). Investigating the two HDL-C groups separately we found a significant association between CETP activity and birthweight in the group with the highest HDL-C; r = 0.19, p = 0.022, but not in the lowest HDL-C group; r = -0.03, p = 0.688.

### Unique lipoprotein composition in women giving birth to SGA infants

**Figure 5:**
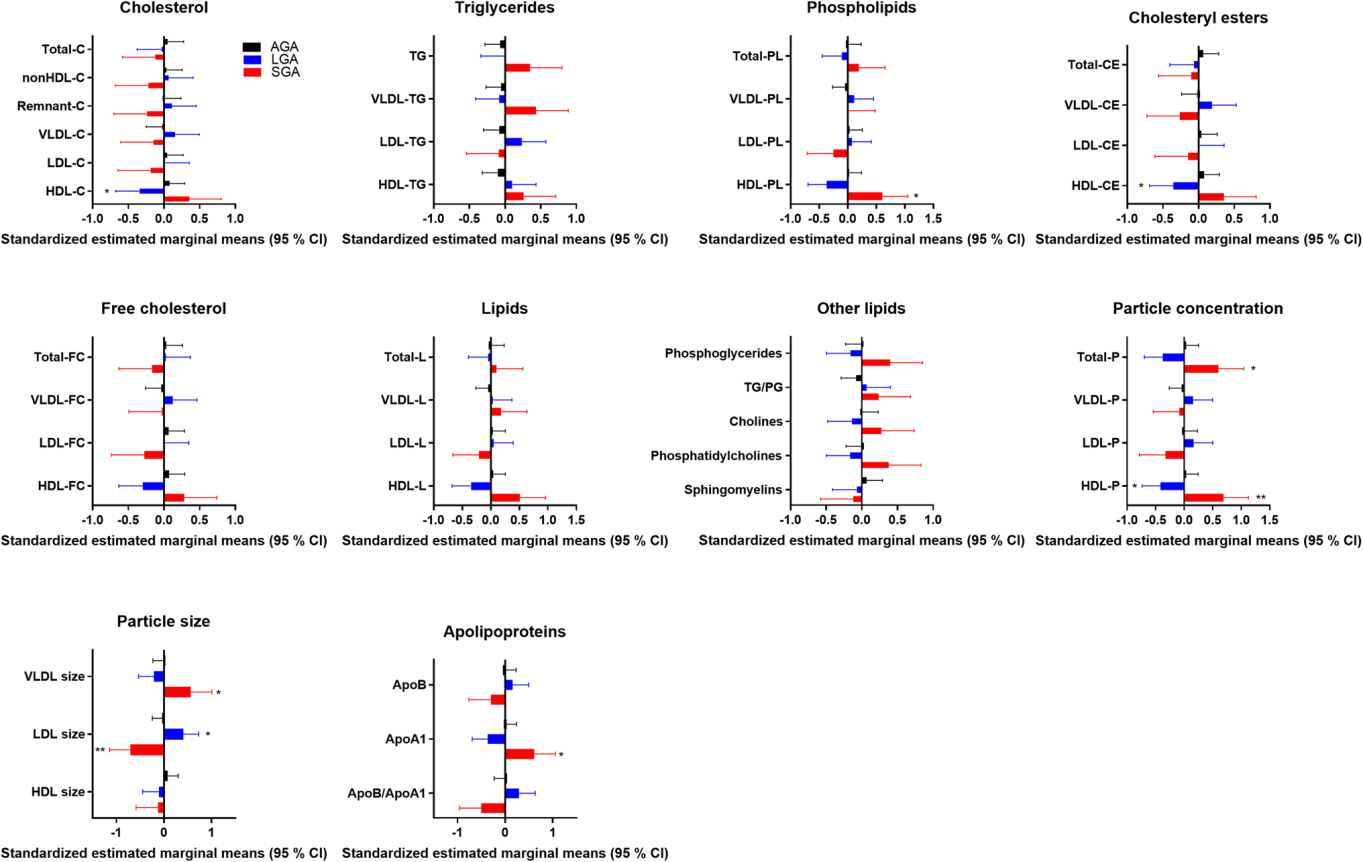
Concentration of total cholesterol, triglycerides, phospholipids, cholesteryl esters, free cholesterol, lipids, particle concentration, particle size at week 36–38 between women giving birth to LGA and SGA vs. AGA infants. Data is normalized and given as standardized estimated marginal means and 95 confidence interval adjusted for age, fetal sex, gestational diabetes, preeclampsia and BMI at week 14–16. *p < 0.05, **p < 0.01, ***p < 0.001.

**Figure 6:**
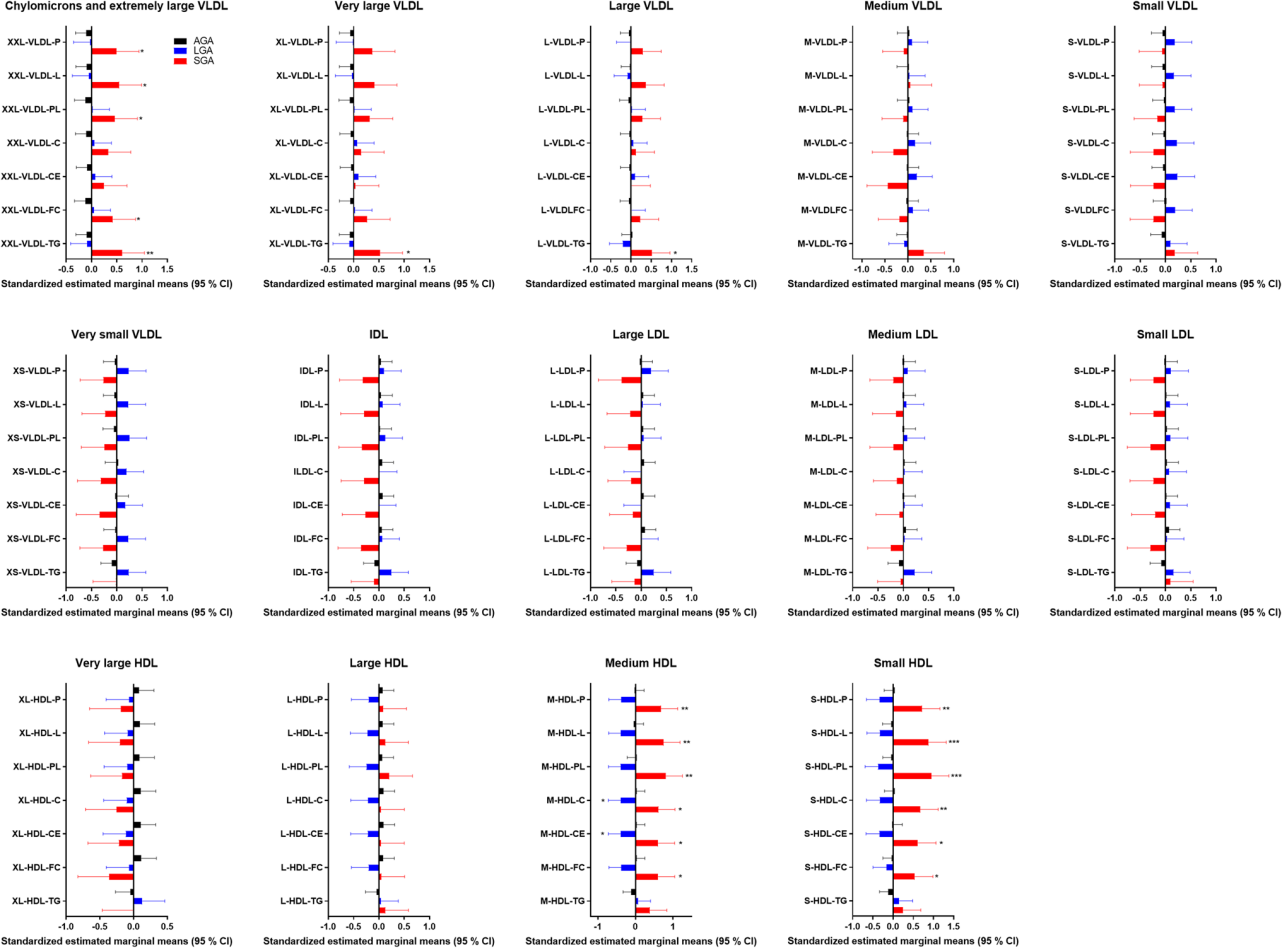
Composition of total (P), lipid (L), phospholipid (PL) cholesterol (C), cholesteryl ester (CE), free cholesterol (FC) and triglycerides (TG) in chylomicrons and very-low-density lipoprotein (VLDL), large density lipoprotein (LDL), intermediate-density lipoprotein (IDL) and high-density lipoprotein (HDL) at week 36–38 between women giving birth to AGA and LGA and AGA and SGA infants. Data is normalized and given as standardized estimated marginal means and 95% confidence interval adjusted for age, fetal sex, gestational diabetes, preeclampsia and BMI at week 14–16. *p < 0.05, **p < 0.01, ***p < 0.001.

**Figure 7:**
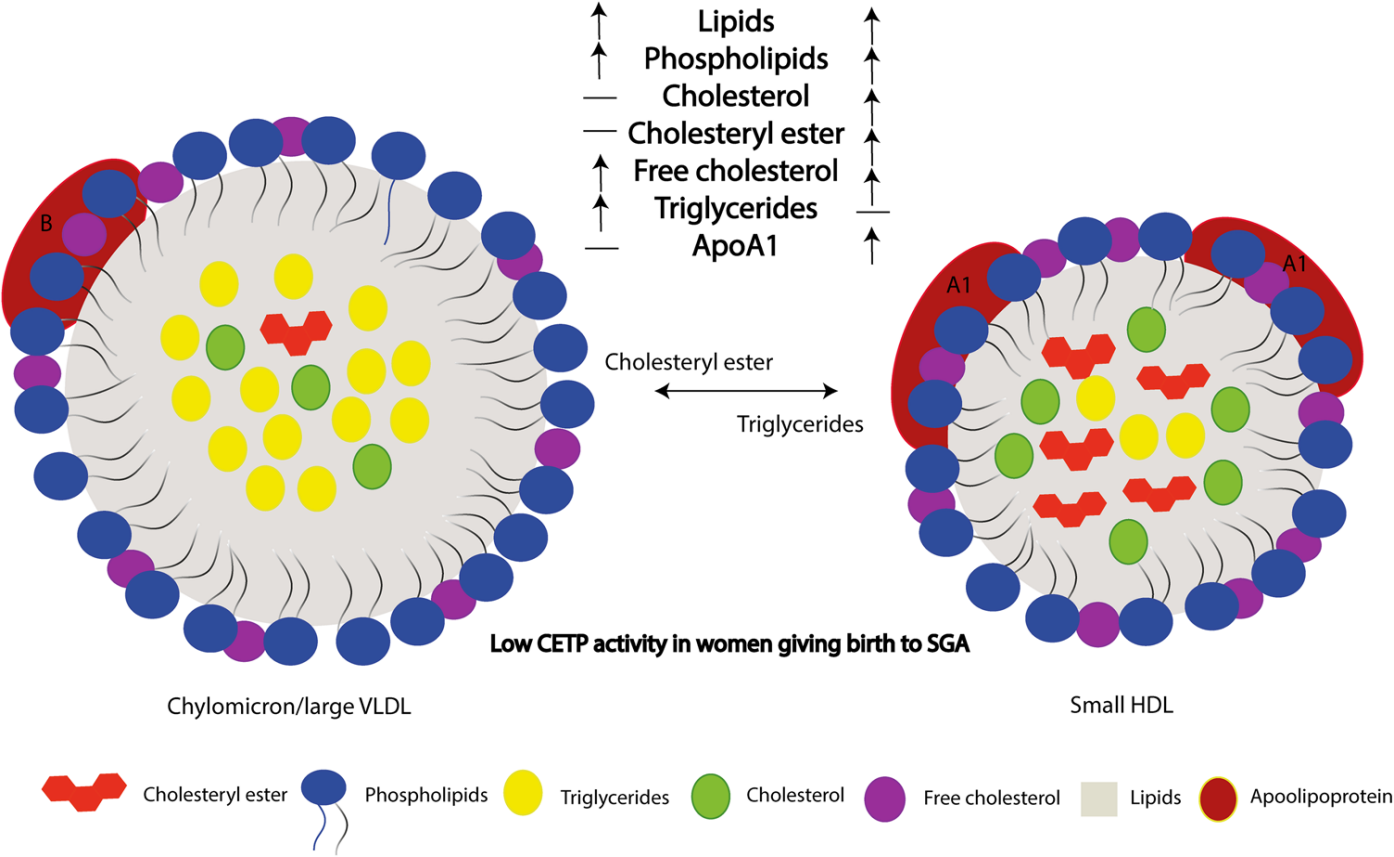
Illustration of the main findings. Low CETP activity in the circulation of women giving birth to SGA infants, followed by low cholesteryl ester and triglyceride exchange between chylomicron/large VLDL and small HDL, resulting in high triglycerides, lipids, phospholipids and free cholesterol in the chylomicrons/large VLDL and high phospholipids, lipids, cholesteryl ester, cholesterol, free cholesterol and ApoA1 in the small HDL compared to the composition in women giving birth to AGA infants.

To get a better understanding of the composition of the lipoproteins in the study group we used a commercially available lipidomics analysis from a subset of the women (n = 160) at weeks 36–38. We found major differences in many of the markers in women giving birth to AGA vs LGA and SGA infants, adjusted for age, fetal sex, GDM, preeclampsia and BMI (Figs. 5, 6). First of all, HDL-C was found lower in the LGA infant group which corresponded to the first analyses of the whole cohort. Briefly, the lipidomics data show that women giving birth to SGA infants have unique composition of the lipoproteins, with high levels of TG in VLDL and high levels of lipids/cholesterol/cholesteryl ester in the medium and small HDL, which corresponds to the low CETP activity (Fig. 7). These analyses also show that women giving birth to SGA infants have higher ApoA1 and HDL particle concentration, with no difference in the size of HDL, showing a unique composition of the medium and small HDL compared to AGA. Moreover, we found that the ABCA1 and ABCG1 in the placenta were associated with CETP in the women with the lower HDL-C but not in the women with the higher HDL-C. Finally, exploring the composition of the lipoproteins in more details (Fig. 6) several interesting findings were revealed. First, we found higher levels of total VLDL, lipids, PL, FC and TG in XXL-VLDL, TG in XL-VLDL and L-VLDL in the SGA infant group. Second and most importantly, total HDL, lipid, PL, cholesterol, CE and FC were higher in M-HDL and S-HDL for the SGA infant group, while cholesterol and CE were lower in M-HDL in the LGA infant group, indicating a unique composition of S-HDL in the women giving birth to SGA infants that are of major importance for the role HDL plays in cholesterol efflux. The main findings are illustrated in Fig. 7.

## Discussion

In the present study we found decreased levels of *CETP* expression in the placentas of women with the highest HDL-C at week 36–38. The *CETP* expression was associated with mRNAs involved in cholesterol/nutrient transport, insulin signaling and inflammation/ECM remodeling. Moreover, both placental and circulating CETP levels were positively associated with growth of the fetus, reflected as increase in AC during third trimester and notably, the regulation of CETP in placenta was strongly related to maternal HDL-C levels. Also, the women giving birth to SGA infants had a lipoprotein composition with high levels of TG in VLDL and lipids/cholesterol/cholesteryl esters in medium and small HDL at week 36–38, corresponding to decreased CETP activity. The modulation of the composition of small HDL particle which also include regulation of ApoA1 may be of particular importance. Finally, low circulating CETP activity during pregnancy at 36–38 weeks was associated with giving birth to SGA infants. Our findings suggest a link between increased maternal HDL-C levels, particularly high cholesteryl ester/cholesterol/lipid in medium and small HDL, low CETP levels both in circulation and placenta and SGA infants, potentially involving altered regulation of metabolic and inflammatory pathways within the placenta.

In pregnancy HDL is present both in the maternal and the fetal circulation. Herein we show that maternal HDL-C level may be a predictor of birth weight and represent a possible link to aspects of placental function. The reduced fetal growth in fetuses from ApoA1^−/−^ mice (low HDL-C) was not mediated by a change in gene expression of ApoA1 or cholesterol synthesis in the fetus itself, and suggested that factors in the maternal circulation were responsible for the change in fetal growth by changing placental function or transport across the placenta affecting the fetus directly^15,16^. In the present study, we show that a large decrease in HDL-C during pregnancy was associated with giving birth to LGA infants, while no decrease in HDL-C during pregnancy was associated with giving birth to SGA infants, compared to a modest decrease in AGA, after adjusting for maternal age and BMI. These data are also supported by previous studies showing increased HDL-C levels at 24–26 weeks gestation in women giving birth to SGA infants^17^. The authors suggest that the elevated HDL-C concentrations were a consequence of placental dysfunction that could diminish the transport of HDL across the placenta to the fetus and thereby raising maternal levels.

Our study shows that women giving birth to SGA infant have higher HDL particle concentration, but with no difference in the size of HDL. However, we found a unique composition of the medium and small HDL compared to AGA. Several studies have found lipid-free ApoA1 and the smaller forms of HDL to be the main acceptors of cholesterol efflux via ABCA1 pathway in macrophages^27^. The cholesteryl ester/lipid rich small HDL in the women giving birth to SGA infants could suggest that the cholesterol efflux is decreased in these women and that they have an unfavorable lipid profile which may in turn have effects on the placenta/fetal growth. Increase in the lipid content of HDL is thought to decrease its capacity to remove cellular cholesterol and small, lipid-poor HDL particles thus represent more efficient cholesterol acceptors^28^. The lipid/cholesterol rich HDL in the maternal circulation may impact the placenta in that also the cholesterol efflux in the placenta is compromised. The cholesterol efflux proteins ABCA1 and ABCG1 control the transport of cholesterol from the maternal circulation to the fetus, and vice versa^29^. Indeed, we found that the ABCA1 and ABCG1 in the placenta were associated with CETP in the women with the lower HDL-C but not in the women with the higher HDL-C. Of relevance to our findings, a decrease of placental ABCA1 expression and cholesterol transport activity has been observed in preeclampsia^30^.

Notably, we found large differences in the mRNA profile of the placenta between mothers with higher and lower HDL-C levels. As a human model to investigate the possible relation of maternal HDL-C levels on mRNA expression in the placenta, we used previous RNAseq data from 28 placentas divided according to maternal HDL-C levels (divided by the median) and investigated differentially expressed genes. mRNAs involved in lipid metabolism and related to inflammation/ECM remodeling were regulated, and we speculate that this could influence placental environment and communication/transport between maternal and fetal interfaces. The inflammation/ECM remodeling genes were mostly upregulated in the placentas of women with low HDL-C, potentially contributing to these women being more metabolic unbalanced which may influence the inflammation/remodeling in the placenta. It is known that the fetal HDL differs from the maternal HDL, however the full HDL proteome in each circulation and its associations is unknown^9^. Interestingly, we found increased expression of *immunoglobulin heavy constant gamma 1 *(*IGHG1*) and *immunoglobulin lambda constant 2 *(*IGLC2*) increased in the placentas of women with the highest HDL-C. These mRNAs have been shown to be part of the fetal HDL proteome, and low abundant in the maternal HDL proteome, and linked to immune response^9^. The impact of the increased levels of these mRNAs is, however, at present unknown. However, investigating the RNAseq data of differentially expressed genes between LGA vs AGA infants we found almost no overlapping mRNAs comparing the list with placenta divided into HDL-C plasma levels, which may suggest that these mRNAs are modulated by HDL-C.

*CETP* was one of the top regulated placental genes and was decreased in the placentas from women with the highest HDL-C group further indicating an interaction between HDL and *CETP.* Placental *CETP* mRNA was also associated with change of AC of the fetus. At the molecular levels, *CETP* expression was associated with regulation of mRNAs involved in cholesterol/nutrient transport, insulin signaling and inflammation/ECM remodeling, and interestingly the association was strongest in the placentas of women with the lowest HDL-C levels. We have previously published adiponectin receptors including *adiponectin receptor 1* (*ADIPOR1)* in the placenta and its association with nutrient transport and fetal growth in LGA infants^31^. Herein we found *CETP* in the placenta negatively associated with *ADIPOR1*. CETP was also associated with mRNAs involved in cholesterol transport including *APOE*, which is a constituent of VLDL and essential ligand for the uptake an clearance of atherogenic lipoproteins and have been found to be secreted to the maternal circulation from the placenta^32^. Further, CETP was associated with mRNAs involved in nutrient transport^33^ including the glucose transporter, *GLUT4*^34^, and mRNAs involved in fatty acid transport^35^. In the liver, *CETP* mRNA levels are increased in response to high cellular cholesterol content^36^ and many binding sites in the CETP promoter have been identified for several transcription factors that regulate its activity^37^. Although our findings could suggest that CETP could be involved in placental-mediated fetal growth, these issues will have to be further clarified as the role of CETP in the placenta is not known. It is a possibility that these changes are driven by HDL-C with CETP as one important mediator.

CETP activity has been shown to be elevated in normal pregnancy compared to non-pregnant individuals^38,39^. Our study includes a larger population and in addition one extra time point closer to term. At week 36–38, we found CETP activity decreased in the whole cohort, and most in the women giving birth to SGA infants. In contrast, a small study found high CETP activity in both maternal and cord sera of SGA infants^40^, while another study found lower CETP mass but a higher cholesteryl ester transfer in neonatal plasma of SGA infants^41^. Surprisingly, in the women giving birth to LGA infants and with the largest decrease in HDL-C during pregnancy, we did not find CETP activity increased during pregnancy, compared to AGA. Further, we did find associations between CETP and change in AC of the fetus, but, these associations were not as strong as for adiponectin in our previous study, where our focus was LGA infants and we found that maternal adiponectin levels are lowest when the fetus has the highest AC. This may suggest that the CETP activity in the maternal circulation is not as important for growth in LGA infants, compared to in the SGA infants. Indeed, we found that CETP activity at 36–38 weeks was associated with SGA infants, and not LGA infants.

Our study has some limitations. Overall, not all markers are measured in all samples and studies are performed on sub-cohorts. The RNAseq was originally not designed for investigating mRNAs that differed in placentas of women with higher and lower maternal HDL-C. These placentas were chosen to investigate differentially expressed mRNAs between placentas from women with preeclampsia and gestational diabetes compared to women without these states. In addition, we did not have any SGA infants in the RNAseq cohort and the small sample size did not allow a proper correction for the several possible confounding factors. Only one biopsy was taken from each placenta and the collection was performed by different individuals. While the technique for placental biopsy was standardized, we cannot exclude the possibility that biopsies were taken at different sites and that regional differences may have accounted for differences in differentially expressed genes. There were too few overlapping samples between RNAseq and the lipidomics data to perform any analysis. Further, we only have CETP activity data of 300 women. Also, we can not exclude some degree of sample degradation before this activity assay, although all samples have been stored in − 80 °C and been treated the same under preanalytical, storage and analytical phase. However, our cohort is a well characterized cohort with clinical data and markers in the blood at several timepoints during pregnancy.

To conclude, our findings suggest a link between increased maternal HDL-C levels, low CETP levels both in circulation and placenta and association with the risk of giving birth to a SGA infant.

## Supplementary Information

Supplementary Information.

## Data Availability

All data generated or analysed during this study are included in this published article (and its Supplementary Information file).

## Abbreviations

AC: Abdominal circumference
CETP: Cholesteryl ester transfer protein
GDM: Gestational diabetes mellitus
HDL: High density lipoproteins
LDL: Low density lipoproteins
LGA: Large-for-gestational age
SGA: Small-for-gestational age
TG: Triglycerides
