# Supplementary material for: Low CETP activity and unique composition of large VLDL and small HDL in women giving birth to small-for-gestational age infants
Source: Sci Rep. 2021 Mar 18;11:6213. doi: 10.1038/s41598-021-85777-3 (PMC7973737; doi:10.1038/s41598-021-85777-3)
Supplement: Supplementary file 1 — Supplementary Information. [file 41598_2021_85777_MOESM1_ESM.docx]

**Supplementary File**

Low CETP activity and unique composition of large VLDL and small HDL in women giving birth to small-for-gestational age infants

Marie Cecilie Paasche Roland, Kristin Godang, Pål Aukrust, Tore Henriksen, and Tove Lekva

**Table 1. A)** mRNAs different in placentas of women with higher and lower HDL-C at week 36-38. Downregulated mRNAs in placentas from women with higher HDL-C levels (shown in grey) and upregulated mRNAs in placentas from women with higher HDL-C levels (shown in white). B) mRNAs different in placentas of women with LGA compared to AGA. Downregulated mRNAs in placentas from women with LGA (shown in grey) and upregulated mRNAs in placentas from women with LGA (shown in white). Excluded low expressed mRNAs and mRNAs not annotated, log2fold>±0.50. Functional annotation in groups (signal, extracellular matrix, calcium, immunoglobulin domain, membrane, polymorphism, endoplasmatic reticulum, lipid transport, myofibrillar myopathy) is included extracted from the bioinformatics database DAVID (<https://david.ncifcrf.gov/>).

| **a) HDL** | | | | | |
| --- | --- | --- | --- | --- | --- |
| Entrez | Log 2 fold | P-value | Symbol | Name | Functional annotation by DAVID |
| ENSG00000111339 | -0,75 | 0,001 | ART4 | ADP-ribosyltransferase 4 (Dombrock blood group) | signal, membrane, P |
| ENSG00000100985 | -0,74 | 0,004 | MMP9 | Matrix metallopeptidase 9 | signal, calcium, ECM, P |
| ENSG00000175084 | -0,72 | <0,001 | DES | Desmin | membrane, MM, P |
| ENSG00000087237 | -0,70 | 0,006 | CETP | Cholesteryl ester transfer protein | signal, LT, P |
| ENSG00000185615 | -0,70 | 0,006 | PDIA2 | Protein disulfide isomerase family A member 2 | signal, ER, P |
| ENSG00000130176 | -0,70 | 0,010 | CNN1 | Calponin 1 | membrane |
| ENSG00000142973 | -0,69 | 0,029 | CYP4B1 | Cytochrome P450 family 4 subfamily B member 1 | membrane, ECM, P |
| ENSG00000105664 | -0,68 | 0,034 | COMP | Cartilage oligomeric matrix protein | signal, ECM, calcium, P |
| ENSG00000152580 | -0,68 | 0,021 | IGSF10 | Immunoglobulin superfamily member 10 | signal, ID, P |
| ENSG00000122679 | -0,68 | 0,024 | RAMP3 | Receptor activity modifying protein 3 | signal, membrane, P |
| ENSG00000101187 | -0,67 | 0,033 | SLCO4A1 | Solute carrier organic anion transporter family member 4A1 | membrane, P |
| ENSG00000163661 | -0,67 | 0,037 | PTX3 | Pentraxin 3 | signal, P |
| ENSG00000086991 | -0,66 | 0,010 | NOX4 | NADPH oxidase 4 | membrane, ER, P |
| ENSG00000186827 | -0,65 | 0,008 | TNFRSF4 | Tumor necrosis factor receptor superfamily, member 4 | signal, membrane, P |
| ENSG00000270816 | -0,65 | 0,031 | LINC00221 | Long intergenic non-protein coding RNA 221 |  |
| ENSG00000111341 | -0,63 | 0,005 | MGP | Matrix Gla protein | signal, P |
| ENSG00000124721 | -0,63 | 0,040 | DNAH8 | Dynein axonemal heavy chain 8 | P |
| ENSG00000087494 | -0,63 | 0,028 | PTHLH | Parathyroid hormone like hormone | signal, calcium, P |
| ENSG00000124406 | -0,62 | 0,034 | ATP8A1 | ATPase phospholipid transporting 8A1 | LT, membrane, ER, P |
| ENSG00000221818 | -0,62 | 0,014 | EBF2 | EBF transcription factor 2 | P |
| ENSG00000188257 | -0,62 | 0,025 | PLA2G2A | Phospholipase A2 group IIA | signal, calcium, membrane, P |
| ENSG00000163687 | -0,61 | 0,018 | DNASE1L3 | Deoxyribonuclease 1 like 3 | signal, ER, P |
| ENSG00000271447 | -0,61 | 0,013 | MMP28 | Matrix metallopeptidase 28 | signal, ECM, calcium |
| ENSG00000173698 | -0,61 | 0,016 | ADGRG2 | Adhesion G protein-coupled receptor G2 | signal, membrane, P |
| ENSG00000169692 | -0,61 | 0,002 | AGPAT2 | 1-acylglycerol-3-phosphate O-acyltransferase 2 | signal, membrane, ER |
| ENSG00000085741 | -0,60 | 0,008 | WNT11 | Wnt family member 11 | signal, ECM, |
| ENSG00000121743 | -0,59 | 0,002 | GJA3 | Gap junction protein alpha 3 | membrane, P |
| ENSG00000109321 | -0,58 | 0,007 | AREG | Amphiregulin | signal, growth factor, membrane, P |
| ENSG00000183615 | -0,58 | 0,005 | FAM167B | Family with sequence similarity 167 member B | signal |
| ENSG00000122367 | -0,57 | 0,046 | LDB3 | LIM domain binding 3 | MM, P |
| ENSG00000273604 | -0,57 | 0,006 | C17orf96 | Elongin BC and polycomb repressive complex 2 associated protein |  |
| ENSG00000143125 | -0,57 | 0,025 | PROK1 | Prokineticin 1 | signal, growth factor, P |
| ENSG00000105808 | -0,56 | 0,009 | RASA4 | RAS p21 protein activator 4 | membrane, P |
| ENSG00000275395 | -0,55 | 0,038 | FCGBP | Fc fragment of IgG binding protein | signal, P |
| ENSG00000143127 | -0,55 | 0,012 | ITGA10 | Integrin subunit alpha 10 | signal, membrane, P |
| ENSG00000123685 | -0,54 | 0,013 | BATF3 | Basic leucine zipper ATF-like transcription factor 3 | P |
| ENSG00000242193 | -0,54 | 0,028 | LOC730102 | Crystallin zeta like 2, pseudogene |  |
| ENSG00000125878 | -0,54 | 0,025 | TCF15 | Transcription factor 15 |  |
| ENSG00000120324 | -0,54 | 0,046 | PCDHB10 | Protocadherin beta 10 | signal, calcium, membrane |
| ENSG00000134198 | -0,53 | 0,024 | TSPAN2 | Tetraspanin 2 | membrane, P |
| ENSG00000133019 | -0,52 | 0,029 | CHRM3 | Cholinergic receptor muscarinic 3 | membrane, P |
| ENSG00000188783 | -0,52 | 0,015 | PRELP | Proline and arginine rich end leucine rich repeat protein | Signal, ECM, P |
| ENSG00000176435 | -0,52 | 0,037 | CLEC14A | C-type lectin domain containing 14A | signal, membrane |
| ENSG00000241644 | -0,51 | 0,023 | INMT | Indolethylamine N-methyltransferase | P |
| ENSG00000149591 | -0,51 | 0,019 | TAGLN | Transgelin | P |
| ENSG00000162998 | 0,51 | 0,038 | FRZB | Frizzled related protein | signal, P |
| ENSG00000154451 | 0,51 | 0,042 | GBP5 | Guanylate binding protein 5 | Membrane, P |
| ENSG00000134247 | 0,52 | 0,042 | PTGFRN | Prostaglandin F2 receptor inhibitor | signal, membrane, ER, ID, P |
| ENSG00000144868 | 0,52 | 0,029 | TMEM108 | Transmembrane protein 108 | signal, membrane, P |
| ENSG00000058335 | 0,54 | 0,047 | RASGRF1 | Ras protein specific guanine nucleotide releasing factor 1 |  |
| ENSG00000164181 | 0,54 | 0,018 | ELOVL7 | ELOVL fatty acid elongase 7 | membrane, ER |
| ENSG00000249464 | 0,54 | 0,020 | LINC01091 | Long intergenic non-protein coding RNA 1091 |  |
| ENSG00000165171 | 0,55 | 0,023 | WBSCR27 | Methyltransferase like 27 | P |
| ENSG00000172575 | 0,57 | 0,035 | RASGRP1 | RAS guanyl releasing protein 1 | calcium, ER, membrane |
| ENSG00000269959 | 0,57 | 0,032 | SPACA6P-AS | SPACA6P antisense RNA |  |
| ENSG00000246145 | 0,57 | 0,005 | RRS1-AS1 | RRS1 antisense RNA 1 (head to head) |  |
| ENSG00000174080 | 0,57 | 0,008 | CTSF | Cathepsin F | signal, P |
| ENSG00000160183 | 0,58 | 0,048 | TMPRSS3 | Transmembrane serine protease 3 | signal, membrane, ER, P |
| ENSG00000091129 | 0,58 | 0,002 | NRCAM | Neuronal cell adhesion molecule | signal, membrane, ID, P |
| ENSG00000231431 | 0,60 | 0,027 | LOC440910 | Uncharacterized LOC440910 |  |
| ENSG00000136235 | 0,60 | 0,022 | GPNMB | Glycoprotein nmb | signal, membrane, P |
| ENSG00000139874 | 0,60 | 0,026 | SSTR1 | Somatostatin receptor 1 | membrane |
| ENSG00000152495 | 0,63 | 0,012 | CAMK4 | Calcium/calmodulin dependent protein kinase IV | calcium, P |
| ENSG00000156475 | 0,63 | 0,005 | PPP2R2B | Protein phosphatase 2 regulatory subunit Bbeta | membrane, P |
| ENSG00000106809 | 0,64 | 0,038 | OGN | Osteoglycin | signal, ECM, growth factor |
| ENSG00000153563 | 0,64 | 0,026 | CD8A | CD8a molecule | signal, membrane, ID |
| ENSG00000147488 | 0,64 | 0,029 | ST18 | ST18 C2H2C-type zinc finger transcription factor | P |
| ENSG00000014257 | 0,64 | 0,001 | ACPP | Acid phosphatase 3 | signal, membrane, P |
| ENSG00000093134 | 0,65 | 0,032 | VNN3 | Vanin 3 | signal, membrane, P |
| ENSG00000113946 | 0,67 | 0,025 | CLDN16 | Claudin 16 | membrane |
| ENSG00000164485 | 0,68 | 0,012 | IL22RA2 | Interleukin 22 receptor subunit alpha 2 | signal, P |
| ENSG00000100336 | 0,68 | 0,001 | APOL4 | Apolipoprotein L4 | signal, membrane, LT, P |
| ENSG00000163377 | 0,70 | 0,030 | FAM19A4 | TAFA chemokine like family member 4 | Signal |
| ENSG00000178031 | 0,71 | 0,011 | ADAMTSL1 | ADAMTS like 1 | Signal, ECM, ID, P |
| ENSG00000164627 | 0,72 | 0,016 | KIF6 | Kinesin family member 6 | P |
| ENSG00000143768 | 0,73 | 0,025 | LEFTY2 | Left-right determination factor 2 | signal, growth factor, P |
| ENSG00000184454 | 0,73 | 0,006 | NCMAP | Non-compact myelin associated protein | membrane |
| ENSG00000178301 | 0,74 | 0,015 | AQP11 | Aquaporin 11 | membrane, P |
| ENSG00000276085 | 0,76 | 0,015 | CCL3L1 | C-C motif chemokine ligand 3 like 1 | signal |
| ENSG00000150471 | 0,77 | 0,005 | ADGRL3 | Adhesion G protein-coupled receptor L3 | signal, calcium, membrane, P |
| ENSG00000249267 | 0,78 | 0,014 | LINC00939 | Long intergenic non-protein coding RNA 939 |  |
| ENSG00000163630 | 0,82 | 0,011 | SYNPR | Synaptoporin | signal, membrane |
| ENSG00000139767 | 0,85 | 0,002 | SRRM4 | Serine/arginine repetitive matrix | P |
| ENSG00000083782 | 0,92 | 0,004 | EPYC | Epiphycan | signal, ECM, P |
| ENSG00000171560 | 0,94 | 0,003 | FGA | Fibrinogen alpha chain | signal, calcium, P |
| ENSG00000138207 | 0,97 | 0,003 | RBP4 | Retinol binding protein 4 | signal |
| ENSG00000166396 | 1,02 | 0,001 | SERPINB7 | Serpin family B member 7 | P |
| ENSG00000211896 | 1,07 | <0,001 | IGHG1 | Immunoglobulin heavy constant gamma 1 | ID, membrane |
| ENSG00000211677 | 1,26 | <0,001 | IGLC2 | Immunoglobulin lambda constant 2 | ID |
| **b) LGA** | | | | | |
| ENSG00000160221 | -0,57 | 0,001 | C21orf33 | Glutamine amidotransferase like class 1 domain containing 3A | signal |
| ENSG00000056291 | -0,54 | 0,002 | NPFFR2 | Neuropeptide FF receptor 2 | membrane |
| ENSG00000145850 | 0,50 | 0,006 | TIMD4 | T cell immunoglobulin and mucin domain containing 4 | signal, ID, membrane |
| ENSG00000156886 | 0,51 | 0,003 | ITGAD | Integrin subunit alpha D | signal, membrane |
| ENSG00000145362 | 0,51 | 0,003 | ANK2 | Ankyrin 2 | membrane |
| ENSG00000213088 | 0,54 | 0,003 | ACKR1 | Atypical chemokine receptor 1 (Duffy blood group) | membrane |
| ENSG00000112149 | 0,55 | 0,002 | CD83 | CD83 molecule | signal, ID, membrane |
| ENSG00000166426 | 0,55 | 0,002 | CRABP1 | Cellular retinoic acid binding protein 1 |  |
| ENSG00000156466 | 0,57 | <0,001 | GDF6 | Growth differentiation factor 6 | signal |
| ENSG00000164949 | 0,59 | 0,001 | GEM | GTP binding protein overexpressed in skeletal muscle |  |
| ENSG00000162706 | 0,60 | <0,001 | CADM3 | Cell adhesion molecule 3 | signal, ID, membrane |

ECM; extracellular matrix, ER; endoplasmatic reticulum, LT; lipid transport, MM; myofibrillar myopathy, ID; immunoglobin domain, P; polymorphism. Some of the mRNAS in the list was not in the output of the DAVID results.
